# The Impact of Stress and Depression on the Outcome of Human Gestation

**DOI:** 10.7759/cureus.48700

**Published:** 2023-11-12

**Authors:** Olga Arvanitidou, Ioannis Kosmas, Christos-Konstantinos Michalopoulos, Martha Doumanidou, Ioanna Ierodiakonou-Benou, Apostolos Athanasiadis, Angelos Daniilidis

**Affiliations:** 1 Family Planning Center, Hippokratio General Hospital, Aristotle University of Thessaloniki, Thessaloniki, GRC; 2 Obstetrics and Gynecology/In Vitro Fertilization and Reproductive Endocrinology, Chatzikosta General Hospital, Ioannina, GRC; 3 Second University Department in Obstetrics and Gynecology, Hippokratio General Hospital, Aristotle University of Thessaloniki, Thessaloniki, GRC; 4 Third University Department of Psychiatry, Aristotle University of Thessaloniki, Thessaloniki, GRC; 5 Third University Department in Obstetrics and Gynecology, Hippokratio General Hospital, Aristotle University of Thessaloniki, Thessaloniki, GRC

**Keywords:** antenatal stress, antenatal depression, preterm birth, birth weight, birth outcomes, antenatal anxiety

## Abstract

Many researchers have reported on the high prevalence of anxiety and depression during pregnancy as well as the influence on delivery outcomes during the past decade. Preterm birth and premature labor, bleeding, higher frequency of cesarean section (CS), low birth weight, preeclampsia, stillbirth, miscarriage, NICU hospitalization, and a low Apgar score are the most commonly referenced outcomes assessed. Clarifying the relationship between exposure and result may help us to understand the risk factors and guide us to future clinical and research practices.

The purpose of this narrative review is to search the following databases: PubMed, Research Gate, Scopus, Medline Plus, and present the most recent, comprehensive literature on the effects of stress and anxiety on pregnancy outcomes. Articles published from 01/01/2000 to 26/11/2022 were obtained from the previous databases.

Anxiety and depression-related disorders are common nowadays, and they are frequently correlated with poor pregnancy outcomes. These problems are caused by a number of factors, including health social determinants, the individual obstetric situation, access to healthcare facilities, etc. The effects of each of these factors on birth outcomes range from major, such as preterm labor, congenital deformities, and low birth weight, to minor, such as mutations in the fetal epigenome.

Both direct and indirect pathways of substantial interactions between depression, anxiety and stress, risk variables, and delivery problems were identified. Women's health practitioners and mental physicians must provide adequate support to these women in order to improve outcomes for both mothers and infants.

## Introduction and background

There have recently been various studies published on the rising incidence of prenatal anxiety and depression. According to recent data, systematic reviews and meta-analyses reported a prevalence of postnatal depression (PND) of 20.7% during pregnancy [1] and 17% in the postpartum period [2].

Several studies have found that maternal anxiety and depression are correlated to greater rates of pre-eclampsia and lower birth weight. Furthermore, depression rates during pregnancy range from 4-20%. Furthermore, recent research has linked perinatal depression to increased spontaneous miscarriage, intrapartum hemorrhage, hypertension, eclampsia, stillbirth, poor Apgar score, low birth weight, newborn development retardation, and greater rates of neonatal intensive care admissions.

## Review

Materials and methods

From 13/09/2022 to 26/11/2022, the authors searched the databases PubMed, Research Gate, Scopus, and Medline Plus for relevant full-text papers published in English. Articles published from 01/01/2000 have been collected. The keywords that were used were antenatal anxiety, antenatal stress, antenatal depression, preterm birth, birth weight, antidepressant, third trimester, and birth outcomes.

Following an initial screening of the title and abstract of all papers, citations considered unnecessary were excluded. The authors extracted the data individually after reading the title and abstract of all possibly eligible papers. When the title and abstract were insufficient to determine the article's eligibility, they examined the whole text. The search was finalized by conducting a manual search of review papers and cross-references. Data submitted solely as abstracts at national and international conferences were also excluded. Patients were not involved in the development of the research question or the outcome measures, nor in the design and implementation of the study, as well as the distribution of the results. Because only published identifying data were evaluated, no Institutional Review Board permission was necessary. No funding was provided to the authors for this work.

Results

**Figure 1:**
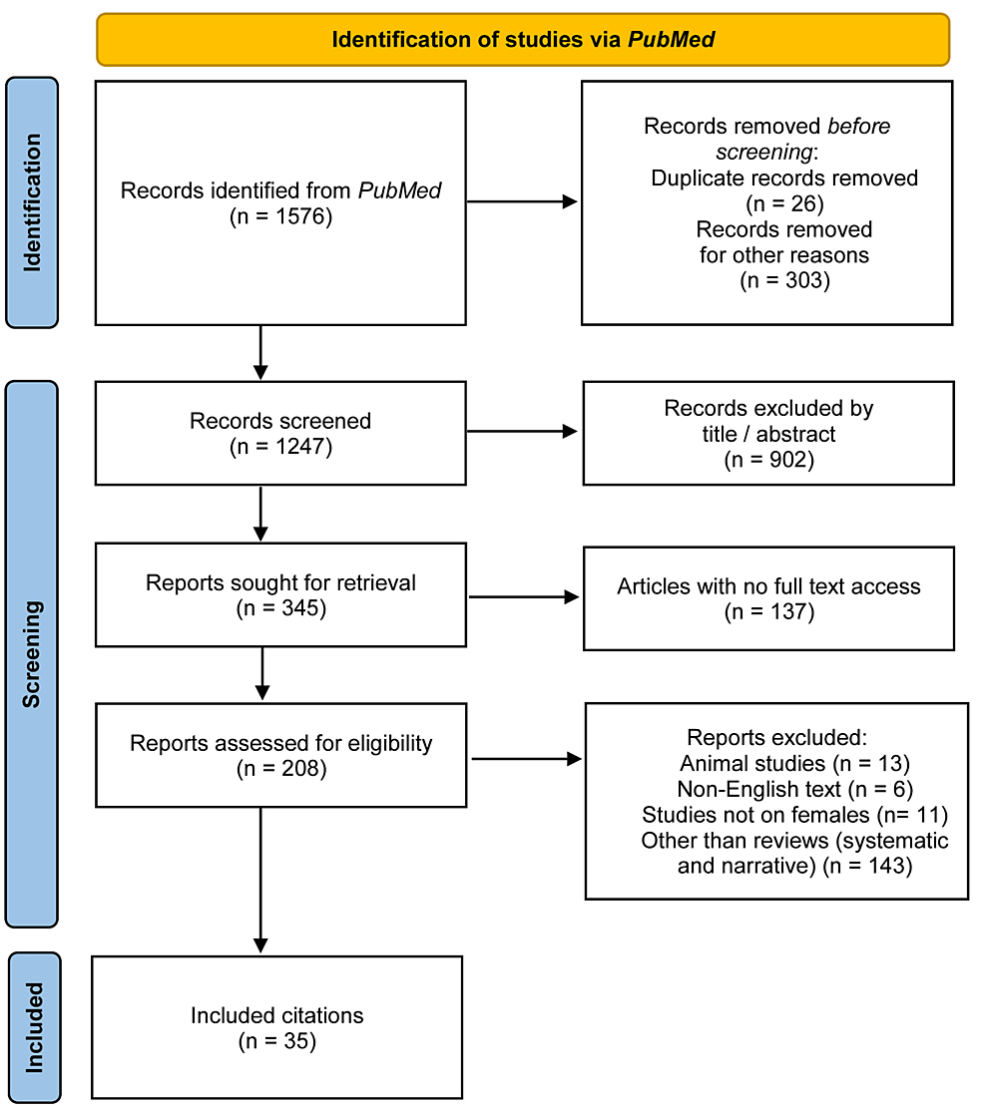
PRISMA flowchart PRISMA: Preferred Reporting Items for Systematic Reviews and Meta-Analyses

A Preferred Reporting Items for Systematic Reviews and Meta-Analyses (PRISMA) flowchart of the study selection is depicted in Figure 1.

Austin et al. released a key research in 2000 revealing that immigrant pregnant women experience high levels of anxiety and stress in the United States (5-10%). By employing preterm labor as a valid indication, the researchers focused on the correlation between antenatal stress and poor obstetric and/or infant outcomes. Multiple studies from the United States in economically underprivileged African American women have found a correlation between perceived maternal life event (LE) stress and premature delivery (~15% in the Black American population). One of the included studies demonstrated that for each point increase on the perceived stress scale, the odds ratio of preterm birth was 1.6 (p=0.003) [3].

Another population-based cohort study published in 2016 by Yu et al. included over 5 million live singleton births followed up to 37 years of age. It was conducted in Denmark (1973-2004) and Sweden (1973-2006) and included mothers who had lost a significant person (child, sibling, parent, and spouse) at the time of their gestation or one year earlier. The findings support that pregnant women who had experienced such a severe loss close to their gestation period were more likely to have poor labor and/or fetal health outcomes, with increased child death rates (3-18%). The second trimester might be the most sensitive period [4].

Yonkers et al. (2009) examined English language papers on prenatal and neonatal outcomes associated with depression and antidepressant medication during childbearing. Depressive disorders of the mother have been linked to an increased incidence of low birth weight (LBW) or small for gestational age (SGA) child delivery(p=0.043). Several studies have found a link between depressive symptoms or a depressive condition and shorter gestations, including post-traumatic stress disorder PTSD. The use of certain antidepressants in early pregnancy is related to an increased risk of spontaneous abortion. Selective serotonin reuptake inhibitors (SSRI) use throughout pregnancy is associated with LBW and SGA reduction. Numerous studies have found that PTD (37 completed weeks of pregnancy) is much higher among women who use antidepressants, particularly SSRIs and tricyclic antidepressants (TCAs). Other research has shown no correlation [5].

Yonkers et al. conducted another prospective cohort analysis of pregnant women between 2005 and 2009, published in 2014. Women who have both post-traumatic stress disorder (PTSD) and a major depressive episode (8% in pregnancy) are four times more likely to have a premature birth; this risk is larger and independent of antidepressant and benzodiazepine use [6].

Dunkel et al. published a review in 2012 demonstrating that chronic stress, racism exposure, and depressive symptoms in mothers during pregnancy are correlated with a low birth weight of the newborn and other negative obstetric outcomes (such as shorter gestation, which has negative consequences for fetal neurodevelopment and child behavior [7].

In a prospective study with 158 women undergoing amniocentesis in the second trimester of pregnancy, Baibazarova et al. examined the association between physiological (cortisol plasma concentration) and self-reported indices (stress, anxiety) of maternal prenatal stress, cortisol in the amniotic fluid, birth outcomes, and infant temperament at the age of three months. Maternal stress and anxiety questionnaire measures were not shown to be correlated to cortisol levels in plasma or amniotic fluid. Maternal cortisol levels were linked to amniotic cortisol levels, which were linked to decreased birth weight (p<0.05) [8].

Lefmann et al. conducted a study in 2014 in which women in Tennessee were divided into two groups: stressed and unstressed. Being in the stressed group did not raise the likelihood of a difficult birth. Having a medical risk or receiving medical aid increased the likelihood of giving birth to premature infants (10.8%) and having a large for gestational age newborn. The findings also showed that young African-American women were less likely to have a difficult birth and that African-Americans had slightly better birth outcomes than European-American women at a younger age [9].

Another prominent research team (Su et al., 2014) proved that when moms encounter stressful events prior to labor, newborns are more likely to have lower birth weights (p=0.007) and smaller head circumferences (p=0.004). Cortisol, adrenocorticotropic hormone (ACTH), norepinephrine, and epinephrine levels were measured at the umbilical cord and linked with birth outcomes of 142 pregnant women. The hormone levels tested were found to be significantly higher, and those children had also been associated with increased rates of poor birth outcomes, such as neuroendocrine system dysfunction and neonatal PTSD (p<0.001) [10].

Another study group by Polanska et al. published an article in 2016 about the impact of maternal stress on various neurodevelopmental outcomes of children during their first two years of life. The findings revealed that maternal stress was the most important factor influencing children's psychomotor development from birth to the age of two years (p=0.01) [11].

A cohort study by El Marroun et al. was conducted in 2017 and 690 children 6 to 9 years old were evaluated to understand how the fetus exposed to maternal and paternal depressive symptoms can affect the formation of brain white matter,-thus directly altering the child's neuronal development until the age of three and even beyond. The children's exposure to maternal depression symptoms during gestation was associated with higher mean diffusivity in the uncinate fasciculus (p=0.05), lower fractional anisotropy (p=0.049), and higher mean diffusivity in the cingulum bundle of their brain [12].

Erickson et al. conducted a review of 34 studies published in 2017 to investigate how prenatal mental health influences the expression and development of children's temperament. While some articles stated that distressed mothers were more likely to have distressed infants, those born by depressed mothers were most likely carriers of faulty serotonin genes. Moreover, motherhood stress was linked to children's irritability (risk 2.41 in mothers with stress vs. 1.9 in mothers without), fearfulness, motor expression, and sadness about 6 months after birth [13].

Cook et al. published a systematic evaluation of 26 studies in July 2017 to integrate and critically analyze quantitative research on the relationship between perinatal PTSD and child outcomes. Maternal postpartum PTSD was found to be correlated with low birth weight and reduced rates of breastfeeding. There is conflicting evidence about the link between maternal PTSD and preterm birth, fetal growth, head circumference, mother-infant interaction, the mother-infant connection, or child development [14].

Malouf and Redshaw conducted a comprehensive evaluation of eleven papers in 2017. The primary goals of the review were to report on the effect of the specialist prenatal clinic on preventing or lowering preterm birth, perinatal mortality, and morbidity. The findings of this review were mixed. There were no differences between normal treatment and care offered at a specialist preterm clinic, according to randomized controlled trials. On the other hand evidence from cohort studies, showed that treatment in specialty clinics for high-risk women is related to a reduction in preterm delivery and lower risks of severe neonatal outcomes [15].

Another research team by Mclean et al., 2018 focused on the relationship that exists between maternal distress during pregnancy and children's anxiety (p<0.01), ignoring fetus discomfort produced by various anatomical/biological characteristics associated with the mother. The researchers noted that children who exhibit anxiety disorders are more likely to do so, due to genetic and/or environmental causes, although additional research is needed [16].

Many studies have found a link between maternal stress and anxiety and pregnancy complications. Data from a longitudinal cohort from Massachusetts General Hospital (Boston), a large tertiary care academic medical center, was used in an observational cohort study. They included women who delivered at 20 weeks of gestation or more in the obstetrics unit at this hospital between 2010 and 2013. The findings demonstrated a link between prenatal depression symptoms and preterm birth (15% of women delivered preterm at less than 37 weeks, 2% very preterm at less than 32 weeks, and 0.2% extreme preterm at less than 28 weeks of gestation) as well as having an SGA neonate at birth [(13%) were SGA at birth, and 8% of neonates were less than 2,500 g] [17].

In China, a prospective longitudinal quantitative study was conducted for 1,470 Chinese pregnant women in two Hong Kong prenatal departments. It included pregnant Chinese-origin women over the age of 18. As an exclusion, pregnant ladies with serious medical conditions were not permitted. This study revealed that eating disorders during pregnancy are strongly linked with higher postpartum disordered eating, higher depression and anxiety symptoms, lower 1-min APGAR score, and abnormal birth weight [18].

Another study using a population-based monitoring system was conducted in Western Hungary. As a result, their database contained 307 participants with participants ranging from 15 to 44 years old. Maternal depression and anxiety had no effect on newborn outcomes. A higher level of maternal self-esteem was linked to greater birth weight and length in boys and greater birth length in girls [19].

In, Spain, a prospective observational research was conducted that included 616 low-risk pregnant nulliparous women ranging from 18 to 42 years old at the time of childbirth. This study found that prenatal education has no effect on childbirth outcomes [20].

One study conducted at two centers in Northern Sweden included 1,495 women who attended two obstetric departments from 2000 to 2001 and found no link between antenatal depression and/or anxiety and pregnancy and delivery complications, such as hypertensive disease, fetal growth restriction, hypoxia and premature deliver, but there was an association with increased health care use (including cesarean sections) during pregnancy and delivery. In this study, 37% of pregnant women, had a sick-leave period at some time during their pregnancy [21].

A prospective study that controls numerous psychological states and sleep quality, reduces the likelihood of correlating delivery fear, anxiety, sleep deprivation, and exhaustion to an increased risk of cesarean section. Enhanced women's childbirth fear may occur as a result of sharing experiences with other multiparous women in processing earlier experiences of difficult and traumatic labor [22].

The findings of this study, which included 331,414 women aged 15 to 45 years old and took place between 1990 and 2009 from a large primary care database in the United Kingdom, revealed that women who had used antenatal medication had a higher risk of all non-live pregnancy outcomes than those who had no history of depression or anxiety. Even some women with preexisting (but untreated) diseases were at elevated risk. When compared to unmedicated women's prenatal morbidity, there was only modest evidence of increased risk in women taking tricyclic antidepressants (TCA's), although there was stronger evidence for several other drugs. In conclusion, women with depression or anxiety have a higher risk of miscarriage, perinatal death, and intention to terminate a pregnancy if they are administered psychiatric medication during early pregnancy than if they are not. Based on these results, it is advisable to avoid or reduce the usage of certain medicines during early pregnancy [23].

Participants in another study were recruited between 1999 and 2002 as part of a prospective study at the Royal Hospital for Women. Antenatal anxiety appears to be a separate risk factor for challenging newborn temperament. This data suggests that antenatal psychological interventions, such as cognitive behavioral therapy, aiming at reducing negative cognitions and anxiety, may be useful in infants' temperament outcomes [24].

Subjects were recruited from all consecutive women attending two prenatal departments in Turin from 2002 to 2005, with a total of 178 mothers examined. The researchers discovered that maternal psychological illnesses are associated with reduced birth weight, although this is unlikely to be owing to aberrant uteroplacental or fetoplacental vascularization [25].

Another systematic review that focused on the association between antenatal depression - anxiety, stress, and preterm birth enrolled all pregnant women, ranging from those clinically diagnosed with depression to those with varying levels of depression, anxiety, or stress, as well as healthy comparator groups. The study's findings indicate an increased overall risk of prematurity when a pregnant woman has one or more of the psychological illnesses specified [26].

This prospective cohort study of 4,408 pregnant women concerned with generalized anxiety disorder (32.6% prevalence) revealed that for a woman experiencing this type of disorder during pregnancy appeared to increase the odds of delivering a low birth weight (adjusted odds ratio (OR)=1.47; 95%CI: 1.10-1.95) or small for gestational age infant (OR=1.39; 95%CI: 1.01-1.92), whereas post-traumatic stress disorder was associated with an increased risk of delivering preterm (OR=1.28; 95%CI: 1.00-1.65). Their findings suggest that antenatal care should provide patients with additional mental health intervention [27].

A cohort study in France contained a consecutive series of 634 pregnant women with singleton pregnancies. The findings of this study revealed that anxiety and depression were associated with particular biological conditions (low BMI, history of preterm labor, and others) and are connected with spontaneous premature labor. In this study, spontaneous preterm birth occurred in 11.4% of women (72/634) [28].

 Another prospective cohort study of 681 women with singleton pregnancies recruited between 20 and 28 weeks of gestation in the obstetrics department of the French University Hospital of Caen. According to the findings of this study, spontaneous preterm birth occurred in 31 women (4.8%). Even after adjusting for potential confounding factors, the rate of this preterm delivery was considerably greater among women with higher depression ratings (9.7%) compared to other women (4.0%). Anxiety was not significantly related to the outcome. The results of this study provide evidence that antenatal depression is significantly linked with spontaneous preterm birth among European women residents [29].

From 2009 to 2010, 219 women with anxiety symptoms living in Israel were recruited for a study. According to the findings, moderate anxiety predicted higher obstetric problems, particularly among mothers of daughters [F(1,109) = 4.56, p = 0.036, ηρ2= 0.06] The same study findings show a group gender interaction on birth weight, with sons of nervous mothers weighing more than sons of controls [F(1,104) = 3.50, p = 0.05, ηρ2 = 0.04] but daughters of anxious mothers weighing less than daughters of the controls [F(1,107) = 4.54, p = 0.035, ηρ2 = 0.04] These findings suggest that mild anxiety symptoms may have an effect on certain birth outcomes, while the findings varied for men and women [30].

Women in low and middle-income nations are subjected to significant psychosocial stress. This study demonstrates that preterm delivery is a common occurrence caused by a variety of etiological factors. Prenatal psychosocial distress (stress, anxiety, and depression), exposure to severe life stressors, and the rate of preterm birth are all of particular relevance when compared to high-income countries [31].

Another study that was carried out at the University of California, between 2000 and 2005, recruited 122 women. 93 women completed the study. There were two groups of women with depression: those who were given antidepressants and those who were not. Their findings revealed that antidepressant use, rather than mild to moderate depression, was associated with a lower gestational age at birth and an increased risk of premature birth. They also discovered that babies born to mothers who were using antidepressants were admitted to the special care nursery at a higher rate. This study found that depression during pregnancy had no negative influence on gestational age at birth [32].

In this review of studies, antidepressant medication use during pregnancy, as well as untreated stress, anxiety, and depression leads to an increased risk of miscarriage, a possible increased risk of congenital cardiac malformations, a higher risk of preterm birth, a small increased risk of persistent pulmonary hypertension of the newborn, and transient neonatal symptoms in about the one-third of neonates. Also, there could be a possible higher risk of delayed motor development in infants [33].

Third-trimester anxiety and depression

In Canada, a longitudinal cohort study, was conducted. Depressive symptoms in the third trimester were shown to be the only mental health characteristic linked with obstetric interventions in this study. Pregnant women with third-trimester depression had an increased chance of emergency cesarean delivery (adjusted odds ratio, 2.04; 95% confidence interval, 1.26-3.29). There was no additional link discovered between antenatal depression and anxiety symptoms and other obstetric treatments. [34]

Another prospective cohort research also included 208 pregnant women. These women were referred to Rasht's Al-Zahra hospital's prenatal clinic in Iran. The study's findings revealed that among participants, changes in pregnancy-specific anxiety are strongly associated with preterm birth. Specific anxiety was not associated with preterm delivery in women in their second trimester, but in their third trimester. [35]

Another cohort study was carried out in Belgrade. They included 83 women with singleton pregnancies with gestational ages ranging from 28 to 41 weeks. This study found that mothers with high trait anxiety had more premature deliveries than those with low or medium anxiety. As a result, maternal prenatal trait anxiety appears to have a deleterious effect on neonatal birthweight [36]

In another prospective cohort study that included 799 pregnant women (55.8% from the East Coast and 44.2% from the West Coast of Malaysia) with mean age of 29.7 (±4.7) years in the third trimester of pregnancy associations of antepartum depressive symptoms with newborn’s low birth weight, preterm birth and cesarean section or instrumental delivery over and above the risk factors of maternal underweight, low income and physical abuse ever, but not antepartum anxiety symptoms. Considering the study sites, the connection of antepartum depressive symptoms with low birth weight is the same in both coasts, antepartum anxiety symptoms become the predictor for both low birth weight and preterm birth in the east coast, and antepartum depressive symptoms for cesarean section or instrumental delivery in the west coast [37].

**Figure 2:**
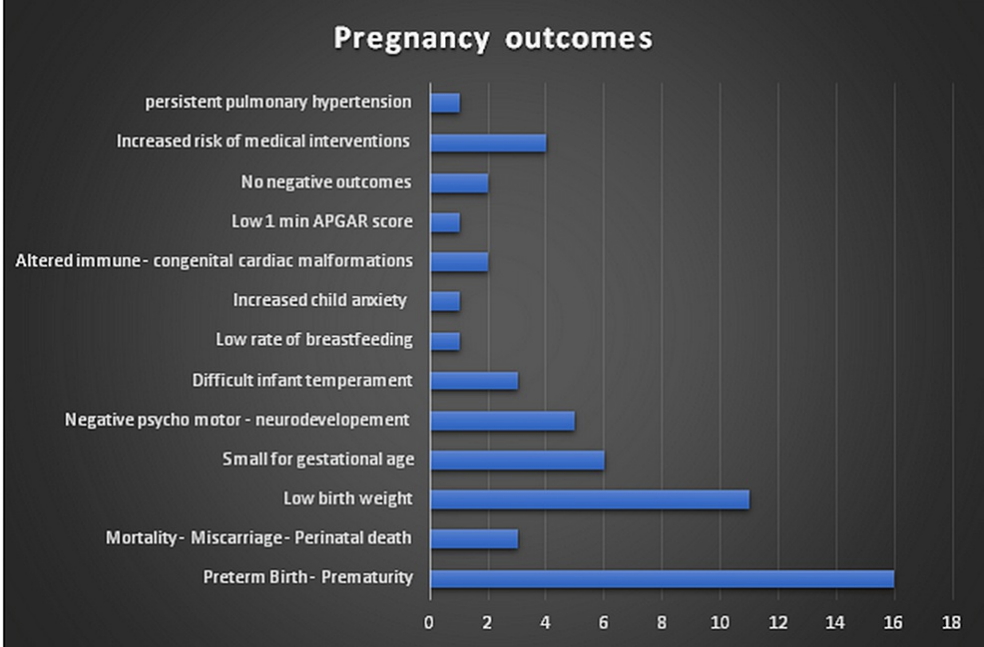
Pregnancy outcomes

**Table 1:** Results of research LBW: Low birthweight, SGA: Small for gestational age, HC: head circumference PPH: persistent pulmonary hypertension

Year of study	Authors	Outcome of stress and anxiety in pregnancy	Type of research
2000	Austin and Leader [3]	Prematurity 3-4% in normal pregnancy	review
2016	Yu et al. [4]	Increased mortality risk by 10%-preterm delivery	review
2009	Yonkers et al. [5]	Prematurity	review
2014	Yonkers et al. [6]	LBW – SGA	cohort
2012	Dunkel et al. [7]	LBW – prematurity – poor fetal neurodevelopment	review
2012	Baibazarova et al. [8]	LBW – infant temperament	cohort
2014	Lefmann TA [9]	Prematurity	review
2014	Su et al. [10]	LBW – smaller HC	review
2016	Polanska et al. [11]	Negative impact to psychomotor development	cohort
2017	El Maroun et al. [12]	Negative impact to psychomotor development	cohort
2017	Erickson et al. [13]	Negative impact to infant temperament	review
2017	Cook et al. [14]	LBW – Lower rates of breastfeeding	review
2017	Malouf and Redshaw [15]	Preterm birth	review
2018	Mclean et al. [16]	Elevated childhood anxiety	review
2018	Vujović et al. [36]	Preterm birth, altered immune and cardio function	cohort
2019	Chan et al. [18]	lower 1-min APGAR score, low birth weigh	cohort
2011	Bödecs et al. [19]	no negative outcomes	cohort
2010	Artieta-Pinedo et al. [20]	no negative outcomes	cohort
2007	Suri et al. [32]	SGA – increased risk of preterm birth	cohort
2004	Andersson et al. [21]	elevated risk of emergency cesarean delivery	cohort
2015	Bayrampour et al. [34]	elevated risk of emergency cesarean delivery	cohort
2012	Hall et al. [22]	increased the risk of using epidural anesthesia	cohort
2012	Ban et al. [23]	miscarriage, perinatal death	cohort
2005	Austin et al. [24]	difficult infant temperament	cohort
2008	Maina et al. [25]	LBW	cohort
2015	Staneva et al. [26]	Preterm birth	review
2020	Gelaye et al. [27]	LBW, SGA, prematurity	Cohort
2019	Nasreen et al. [37]	LBW, preterm birth, cesarean section,	cohort
2018	Khalesi and Bokaie [35]	Preterm birth	cohort
2002	Dayan et al. [28]	Preterm birth	cohort
2006	Dayan et al. [29]	Preterm birth	cohort
2014	Kaitz et al. [30]	LBW	cohort
2015	Premji et al. [31]	Preterm birth	review
2016	Venkatesh et al. [17]	SGA, preterm birth	cohort
2015	Pearlstein T [33]	PTB, PPH, delayed motor development, miscarriage, congenital cardiac malformations	review

In our studies, the most common type of research was cohort study (22, 62.85%) followed by reviews (12, 37.14%). The results of our study showed that preterm birth (prematurity) was the most probable negative outcome of all, with 16 out of the 35 studies reviewed mentioning it (45.71%), followed by low birth weight infants with 11 studies (31.42%). Small for gestational age infants come third with six studies referring to it (17.14%.) Next in our study comes the negative development of psychomotor and neurodevelopment with five outcomes (14.28%), increased risk of medical interventions (cesarean section, epidural anesthesia, etc.) with four mentions (11.42%), and mortality - miscarriage - prematurity equals with difficult infant temperament with three referrals ( 8.57%). Another two studies revealed that an altered immune system and congenital cardiac malformations are negative outcomes of prenatal depression and anxiety (5.71%). There were also two studies in our research that had no negative pregnancy outcome (5.71%). Increased child anxiety, low 1-minute Apgar score, persistent pulmonary hypertension, and low rate of breastfeeding were all mentioned in one study (2.86%) (Figure 2 and Table 1).

## Conclusions

Many studies have found that anxiety, depression, and PSTD increase the risk of adverse birth outcomes (preterm labor, lower birth weight, and lower Apgar score). Several years after labor, the children's emotional, neurological, and motor development appear to be strongly correlated with the mother's well-being, mental health status, and socioeconomic conditions during the perinatal period, highlighting the need for more and better women's support from health care providers. The significance and necessity of further research in the future are emphasized, particularly in investigating the relationship and impact of anxiety and depression on fetal development.
